# Multilevel synchronization of human *β*-cells networks

**DOI:** 10.3389/fnetp.2023.1264395

**Published:** 2023-09-22

**Authors:** Nicole Luchetti, Simonetta Filippi, Alessandro Loppini

**Affiliations:** ^1^ Center for Life Nano and Neuro-Science, Istituto Italiano di Tecnologia, Rome, Italy; ^2^ Engineering Department, Università Campus Bio-Medico di Roma, Rome, Italy; ^3^ National Institute of Optics, National Research Council, Florence, Italy; ^4^ International Center for Relativistic Astrophysics Network, Pescara, Italy

**Keywords:** functional networks, multiplex, metabolic coupling, calcium wave, bursting, slow oscillations, islets, hubs

## Abstract

*β*-cells within the endocrine pancreas are fundamental for glucose, lipid and protein homeostasis. Gap junctions between cells constitute the primary coupling mechanism through which cells synchronize their electrical and metabolic activities. This evidence is still only partially investigated through models and numerical simulations. In this contribution, we explore the effect of combined electrical and metabolic coupling in *β*-cell clusters using a detailed biophysical model. We add heterogeneity and stochasticity to realistically reproduce *β*-cell dynamics and study networks mimicking arrangements of *β*-cells within human pancreatic islets. Model simulations are performed over different couplings and heterogeneities, analyzing emerging synchronization at the membrane potential, calcium, and metabolites levels. To describe network synchronization, we use the formalism of multiplex networks and investigate functional network properties and multiplex synchronization motifs over the structural, electrical, and metabolic layers. Our results show that metabolic coupling can support slow wave propagation in human islets, that combined electrical and metabolic synchronization is realized in small aggregates, and that metabolic long-range correlation is more pronounced with respect to the electrical one.

## 1 Introduction

Endocrine *β*-cells activity is crucial for regulating blood glucose levels, as well as lipid and protein metabolism, through coordinated bursting oscillations triggering a calcium-driven insulin release (Dimitriadis et al., 2011; Rorsman et al., 2011; Rorsman and Braun, 2013). In the mouse, coordination of electrical and calcium oscillations has been shown to be primarily determined by gap-junction coupling, as demonstrated by both experimental (Ravier et al., 2005; Speier et al., 2007; Benninger et al., 2008; Head et al., 2012) and modeling studies (Sherman and Rinzel, 1991; Smolen et al., 1993; Bertram et al., 2000; De Vries and Sherman, 2000; Aslanidi et al., 2002). In this regard, electrical coupling permits smoothing *β*-cells stochasticity and heterogeneity, with important effects in the appearance of coordinated electrical oscillations, in optimizing bursting oscillation period, in supporting activation waves within pancreatic islets, and thus in ensuring an effective pulsatile insulin release. Other than electrical coordination, gap-junction coupling was also shown to permit metabolites diffusion through neighboring cells (Kohen et al., 1979; Rao and Rizzo, 2020), further allowing metabolic synchronization. However, metabolic coupling in pancreatic islets was only partially investigated (Tsaneva-Atanasova et al., 2006; Loppini and Chiodo, 2019), and its role in islet functioning has yet to be fully grasped. In this regard, Tsaneva-Atanasova et al. (2006) theoretically proved that G6P diffusion through gap junctions can switch slow bursting into fast one through oscillation death of the slow metabolic component, while Loppini and Chiodo (2019) showed that metabolic coupling promotes slow bursting coordination in human-like *β*-cells clusters, and electrical correlation is spatially extended by the inclusion of a subpopulation of cells with increased metabolically activity. Another crucial aspect is that the role of gap junctions in *β*-cells aggregates was mostly studied in the mouse, while in human islets, which differ in composition and cells spatial arrangement from mouse (Brissova et al., 2005; Cabrera et al., 2006; Steiner et al., 2010), only a few studies explored the role of coupling in emergent *β*-cells activity (Loppini et al., 2015; Loppini et al., 2017; Loppini and Chiodo, 2019). In this context, approaches combining mathematical modeling and electrophysiological recordings from small human *β*-cells aggregates showed that human gap-junction connections are characterized by similar electrical conductance with respect to the mouse and are still crucial in promoting synchronization and enhanced fast bursting oscillation periods. The lack of comprehensive investigations on metabolic coupling, and, in general, on both electrical and metabolic coupling in human islets, is also more relevant if we take into account recent studies based on calcium imaging that has further enriched the picture of *β*-cells aggregates coordination (Stožer et al., 2013a; Markovič et al., 2015; Johnston et al., 2016; Gosak et al., 2018; Salem et al., 2019; Šterk et al., 2023b). Indeed, it was shown that *β*-cells functional networks, as derived from correlation indices computed on cells activity, are heterogeneous, and characterized by hub cells with an increased number of functional connections with respect to the others, coordinating the response of other cells and ensuring whole-islet synchronization. This peculiar sub-type of cells was also shown to have increased metabolic activity.

In this contribution, we explore the combined role of electrical and metabolic coupling in human *β*-cells networks through a biophysically detailed mathematical model, analyzing spatiotemporal electrical and metabolic emerging activities and describing whole-islet synchronization through functional networks. Specifically, simulated signals are used to derive electrical and metabolic functional layers that are further studied in comparison to the underlying islet cytoarchitecture, i.e., the structural layer, in a three-layer multiplex network, in line with previous experimental studies analyzing membrane potential and intracellular calcium dynamics in mouse islets (Gosak et al., 2015). We analyze *β*-cells coordination patterns by looking at the multiplex pairwise connection motifs, analyzing functional connections on the electrical and metabolic layers and their occurrence in comparison to gap-junction mediated structural couplings. Further, we investigate the possible effects of biological noise and double-population heterogeneity on emergent coordination.

## 2 Materials and Methods

### 2.1 *β*-cell networks modeling

We built our biophysical model of human *β*-cell networks based on a comprehensive electrophysiological description of human *β*-cells activity, also accounting for metabolites oscillations (Pedersen, 2010; Riz et al., 2014; Loppini et al., 2015), and added gap-junction coupling between adjacent cells, defined by the islet structure as detailed in the next subsection.

The equation for the membrane potential dynamics of the *i*th cell is

$$\frac{dV_{i}}{dt}=-\left(I_{\text{ion,}i}+\underset{j\in \Omega_{i}}{\sum }I_{\text{coup,}ji}\right),$$ (1)

where *I*_ion,*i*_ is sum of the membrane ionic currents, Ω_*i*_ is the cell neighborhood and *I*_coup,*ji*_ = *g*_c_(*V*_*i*_ − *V*_*j*_) is the gap-junction current between *i* ^th^ and *j* ^th^ cells.

Among the ionic currents included in the electrical compartment, the ATP-sensitive potassium (KATP) current is modulated by a variable mimicking the slow periodic ATP increasing and decreasing downstream of the glycolytic oscillations, triggering action potential firing and bursting. The glycolytic compartment, models the dynamics of intermediate metabolites whose production follows the glucose uptake by the cell, i.e., glucose 6-phosphate (G6P), fructose 6-phosphate (F6P), fructose 1,6-bisphosphate (FBP), dihydroxyacetone-phosphate (DHAP) and glyceraldehyde 3-phosphate (G3P). In our model, the metabolic coupling between nearby cells is realized imposing G6P and F6P diffusion through gap junctions. In particular, the dynamical equation regulating the total concentration of G6P and F6P (G6PF6P) for the *i* ^th^ cell is

$$\frac{{d\left[G6PF6P\right]}_{i}}{dt}=V_{\text{GK},i}-V_{\text{PFK}}-P_{\text{G}6\text{PF}6\text{P}}\underset{j\in \Omega_{i}}{\sum }\left({\left[G6PF6P\right]}_{i}-{\left[G6PF6P\right]}_{j}\right),$$ (2)

where *V*_GK_ and *V*_PFK_ are the glucokinase and phosphofructokinase reaction rates, and *P*_G6PF6P_ denotes the gap-junction permeability to metabolites, i.e., the strength of metabolic coupling. As reference case, we set the electrical conductance and metabolites permeability of gap junctions to *g*_c_ = 0.01 nS/pF and *P*_G6PF6*p*_ = 0.01 ms^−1^, in line with previous estimations based on electrophysiological data and experimental measures of metabolites diffusion among adjacent *β*-cells (Kohen et al., 1979; Loppini et al., 2015). These values are also used in other modeling studies on *β*-cells networks (Loppini et al., 2015; Loppini et al., 2017; Loppini and Chiodo, 2019; Saadati and Jamali, 2021). Biological heterogeneity among cells was reproduced by varying the conductance of the voltage-sensitive potassium channels (*g*_Kv_) for the electrical compartment and *V*_GK_ for the metabolic one, keeping the other parameters fixed at their default values (Loppini et al., 2015). In particular, *g*_Kv_ can impose a very fast busting or spiking response with variable frequency, and *V*_GK_ sets the intrinsic metabolic oscillation frequency. In order to simulate a fast spiking activity within the slow bursting active period, we set mean value and standard deviation of *g*_Kv_ to 0.215 nS/pF and 2%, respectively, while for *V*_GK_ to 0.0556 mM/s and 10%. The effect of these parameters was tested in uncoupled populations and single-cell simulations. This allowed for the identification of a *V*_GK_ cut-off value setting the onset of metabolic oscillations. Low values of *V*_GK_ not inducing metabolic oscillations at the single-cell level were discarded.

A full list of model equations, their detailed description and the complete set of parameters can be found in refs. Riz et al. (2014), Loppini et al. (2015).

### 2.2 Islet architecture

It is known that the spatial organization of *β*-cells in pancreatic islets and islets composition differ across species (Brissova et al., 2005; Cabrera et al., 2006; Steiner et al., 2010). In humans, *β*-cells appear to be intermingled with *α*-, *γ*-, and *δ*-cells, and are about 50% of the islet cells. We built human-like *β*-cells networks by stacking 2D layers of cells in a hexagonal-closest packing (HCP) structure (Nittala et al., 2007; Nittala and Wang, 2008; Stamper et al., 2014; Cappon and Pedersen, 2016), with 12 layers, each including 12 × 12 cells. A single cell was modeled as a sphere of radius *r*_c_ = 6.5 *μ*m. To build the final islet structure, we extracted a spherical region with 65 *μ*m radius within the HCP stacked layers and imposed a site percolation probability of 50% (p_site_ = 0.50) to match data on *β*-cell percentage. Nearest-neighbor couplings were defined based on structural contacts between cells, i.e., for cells whose center-to-center distance was equal to 2*r*_c_. We further deleted from the structure isolated cells resulting from percolation (less than 5). The total number of cells included in the reconstructed islet was *N* = 378, with an average and maximum number of neighbors equal to ≃ 5 and 10, respectively. The structural adjacency matrix *a*_*ij*_ describing nearest-neighbors was then used in model equations to define the electrical and metabolic coupling terms (Eqs 1, 2).

### 2.3 Functional network and motif analysis

Synchronization phenomena have been investigated by computing the correlation index between all pairs of *β*-cell binarized potential and metabolic signals (Rodgers and Nicewander, 1988; Stožer et al., 2013b):

$$R_{ij}=\frac{\left\langle \left(x_{i}\left(t\right)-\left\langle x_{i}\left(t\right)\right\rangle \right)\left(x_{j}\left(t\right)-\left\langle x_{j}\left(t\right)\right\rangle \right)\right\rangle }{\sigma_{i}\sigma_{j}},$$ (3)

where *x*_*i*_ and *σ*_*i*_ are the binarized signals and the corresponding standard deviation for the *i* ^th^ cell. The binarized signals were constructed by evaluating at first the onset time in cells activation, both on membrane potential (*V*) and FBP, by means of a signal derivative thresholding, and at second, by imposing an active time duration of 15 ms for *V*, and 1.2 s for FBP. Therefore the binarized signals were equal to 0 and 1 outside and inside the active time, respectively. This choice allowed us to match the action potential duration in *V* and the initial rise in FBP.

The correlation indices were further thresholded to obtain electrical and metabolic functional networks. In particular, we investigated the behavior of the networks by varying the correlation thresholds, R_V,thr_ and R_FBP,thr_, in the range of [0,1]. We finally performed a motif analysis by considering, at the three network levels (structural, electrical, and metabolic), the pairwise multiplex connection motifs, for a total of 2^3^ = 8 possible motifs. In this context, a multiplex pair-wise connection motif represents how *β*-cell pairs are connected through the structural, electrical and metabolic layers, and serves to compare cells connectivity at different levels. We investigated connection motifs at increasing distances over the islet, quantifying synchronization spatial features in comparison to the underlying structural architecture. To choose the optimal value of the correlation thresholds to be used in the motif analysis, we studied four network parameters (average node degree, average local clustering coefficient, average local efficiency, and number of connected components) as functions of the correlation thresholds for both electrical and metabolic dynamics. Specifically, the average node degree is evaluated as the mean of the nodes degree *k*_*i*_ = *∑*_*j*∈*N*_ *a*_*ij*_. The average local clustering is the mean of the local clustering coefficient of the nodes *c*_*i*_ = 2*e*_*i*_/(*k*_*i*_(*k*_*i*_ − 1)), where *e*_*i*_ is the number of connections in the local subgraph of node *i*, i.e., *G*_*i*_. Similarly, the average local efficiency is the mean of the local efficiency of the nodes $E_{i}=\left(1/\left(k_{i}\left(k_{i}-1\right)\right)\right)\sum_{j\neq j^{\prime }\in G_{i}}1/d_{jj^{\prime }}$ , where *d*_*jj*′_ is the distance between nodes *j* and *j*′ (Latora and Marchiori, 2001; Boccaletti et al., 2006). In relation to intercellular networks, the node degree quantifies the number cells to which a cell is connected, the local clustering coefficient measures the tendency of cells to develop dense local connection patterns, the local efficiency is linked the clustering coefficient and quantifies how efficient is the local intercellular communication, while the number of connected components measures the amount of disjoint connected cells groups in the network. In the context of functional networks, high degree, clustering and efficiency, and a low number of connected components denote strong correlations in cells activity encompassing large groups of cells.

### 2.4 Numerics

*β*-cells architecture was reproduced with MATLAB R2021a (The MathWorks, Inc.), together with the data analysis. The numerical integration of the biophysical model is implemented in a C++ algorithm using a fourth-order Runge–Kutta scheme for the electrical subsystem, and an Euler scheme for the metabolic subsystem in parallel, due to the non-restrictive slow dynamics of the glycolytic oscillator. In each case, a fixed time step of 0.02 ms was used. Numerical accuracy was verified with a MATLAB implementation for both the electrical and the glycolytic compartments, using a stiff integrator.

## 3 Results

### 3.1 Electrical and metabolic coupling: bursting and activation waves

**FIGURE 1:**
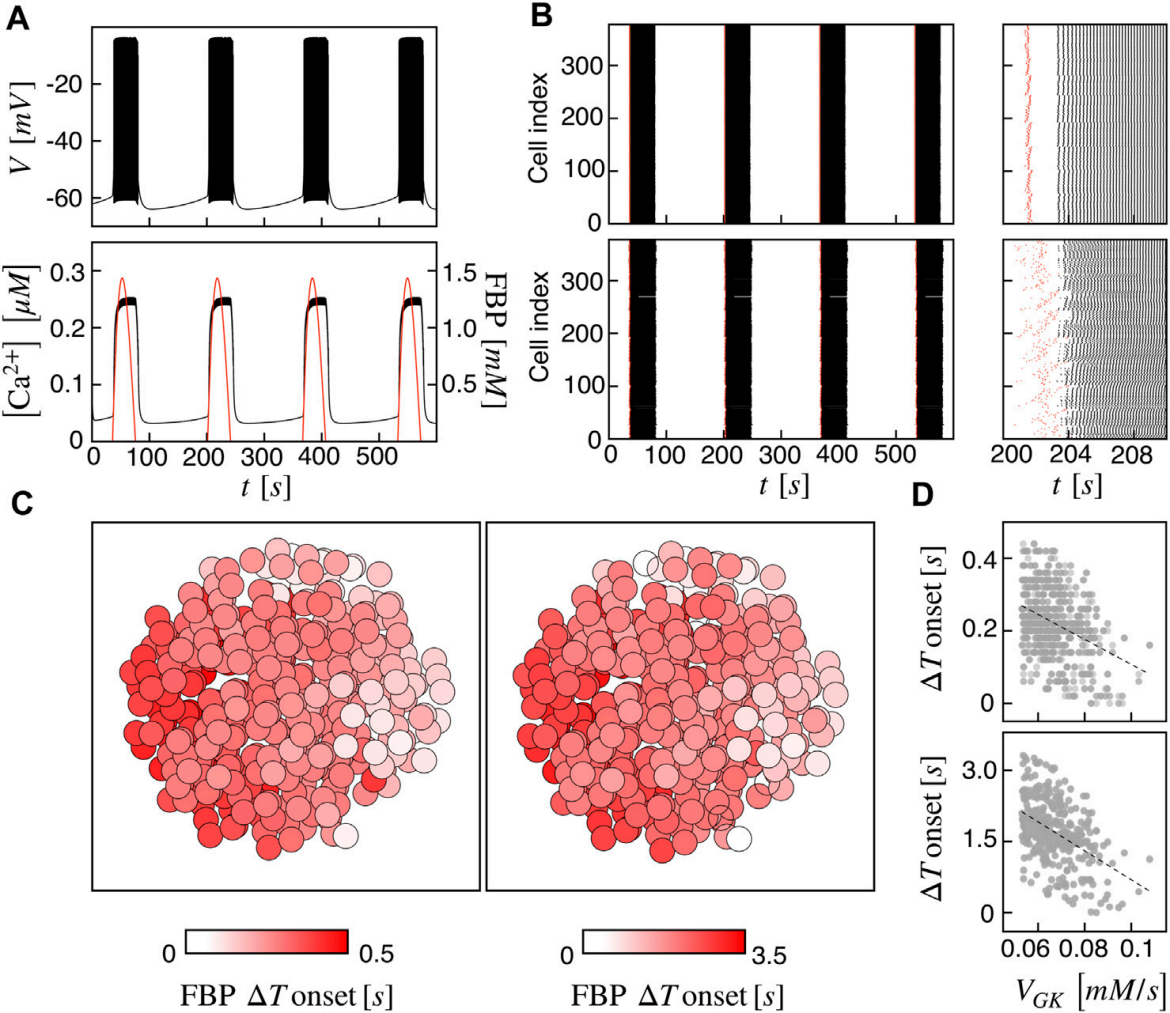
Spatiotemporal islet dynamics. **(A)** Simulated membrane potential (top), calcium (bottom, black curve) and FBP (bottom, red curve) signals for a reference cell, computed at *g*_c_ = 0.01 nS/pF and *P*_G6PF6P_ = 0.01 ms^−1^. **(B)** Raster plots of cells activation times for membrane potential *V* (black) and FBP (red) at different coupling strengths: *g*_c_ = 0.01 nS/pF and *P*_G6PF6P_ = 0.01 ms^−1^ (top), *g*_c_ = 0.01 nS/pF and *P*_G6PF6P_ = 0.001 ms^−1^ (bottom). Insets on the right show zooms of a single activation front. **(C)** Average activation pattern mapped on the islet structure, showing the FBP delay of activation with respect to the first responding cell (FBP Δ*T* onset) in color code, all over the islet and at different G6PF6P permeability values: *P*_G6PF6P_ = 0.01 ms^−1^ (left, model I1), *P*_G6PF6P_ = 0.001 ms^−1^ (right, model I2). **(D)** Correlation between FBP Δ*T* onset values and the glucokinase reaction rate *V*_GK_ over the *β*-cells population for both I1 (top) and I2 (bottom). Dashed lines correspond to linear fits highlighting the inverse proportionality between the two quantities.

At first, we investigated the islet dynamics with electric and metabolic coupling strengths as previously used in other modeling studies, i.e., *g*_c_ = 0.01 nS/pF and *P*_G6PF6P_ = 0.01 ms^−1^. At the single-cell level, the pattern is a slow bursting oscillation driven by glucose metabolism with a bursting period of ≃ 160 s (frequency of ≃ 0.37 min^−1^) and active phase duration of ≃ 45 s, resembling slow electrical activity observed in human *β*-cells (Braun et al., 2012; Rorsman and Braun, 2013; Riz et al., 2014). The membrane potential is characterized by a fast action potential firing intermingled to silent periods, while the intracellular calcium shows a compound oscillation with fast and slow components following the membrane potential oscillations and metabolites dynamics, respectively (Figure 1A). As a representative variable of the glycolytic component, we focused on FBP, whose oscillations are in phase with membrane potential active phases and with the slow calcium component. To analyze cells coordination at the population level, we investigated the activation times of cells on both membrane potential and FBP, as extracted from the peaks of signals derivative. We observed a coherent and synchronized activity all over the islet, characterized by in-phase action potentials within the active periods both between near and distant *β*-cells. Dephasing in cells activity was instead observed at the onset and termination of the active phases. Indeed, cells activation at the beginning of the active phases followed a wave pattern propagating through the islet. This pattern was observed on membrane potential, on calcium, and on FBP and was progressively lost when moving within the central part of the active phases, where cells were more synchronized and electrically activating in small groups. The wave pattern and the synchronized regime just after are shown in Figure 1B (top) by means of raster plots computed on the FBP and membrane potential activation times. To further dissect the spatial organization of the activation wave, we evaluated, for each burst, the FBP activation time for every cell with respect to the first responding cell in the islet (Δ*T* onset) and analyzed its spatial spreading over the islet structure (Figure 1C, left). At *g*_c_ = 0.01 nS/pF and *P*_G6PF6P_ = 0.01 ms^−1^, Δ*T* onset was in the range 0–0.45 s with an organized spatial distribution indicating an activation wave triggered by peripheral cells and spreading toward the opposite area of the islet. To get deeper insights in wave dynamics, we analyzed the correlation of Δ*T* onset versus the values of parameters we varied in the *β*-cells population (*V*_GK_ and *g*_Kv_) and found a negative correlation with the glucokinase reaction rate *V*_GK_ (Figure 1D, top), suggesting that the wave initiators were also among the cells with increased glucokinase reaction rate. Given that the diameter of our simulated islet was ≃ 125 *μ*m, we estimated a wave velocity of about 280 *μ*m/s at this coupling conditions, a value significantly higher than the ones experimentally measured in real human islets, close to ≃ 10 *μ*m/s (Gosak et al., 2022). Based on this aspect, we further tested the model at a lower metabolites permeability, i.e., at *P*_G6PF6P_ = 0.001 ms^−1^, keeping unaltered the electrical coupling. In this conditions, same bursting dynamics was recovered at the single-cell level, but at the population level, cells dephasing in slow oscillations was more pronounced, especially at bursting onset (Figure 1B, bottom). We observed an increasing in Δ*T* onset, ranging in this case between 0 and 3.5 s. Also, its spatial distribution followed a similar organization as the one computed at higher metabolites permeability, still indicating a wave spreading phenomena triggering the islet activation (Figure 1C, right). Same anti-correlation between Δ*T* onset versus *V*_GK_ was computed, as for the high permeability case (Figure 1D, bottom). Interestingly, in this condition we could obtain a wave velocity of ≃ 36 *μ*m/s, i.e., on the same order of the one measured experimentally. In the following, the functional network analysis is performed on both models with high and low permeability to G6PF6P. We refer to the high and low permeability islet models as I1 and I2.

### 3.2 Electrical and metabolic functional networks: multiplex description

**FIGURE 2:**
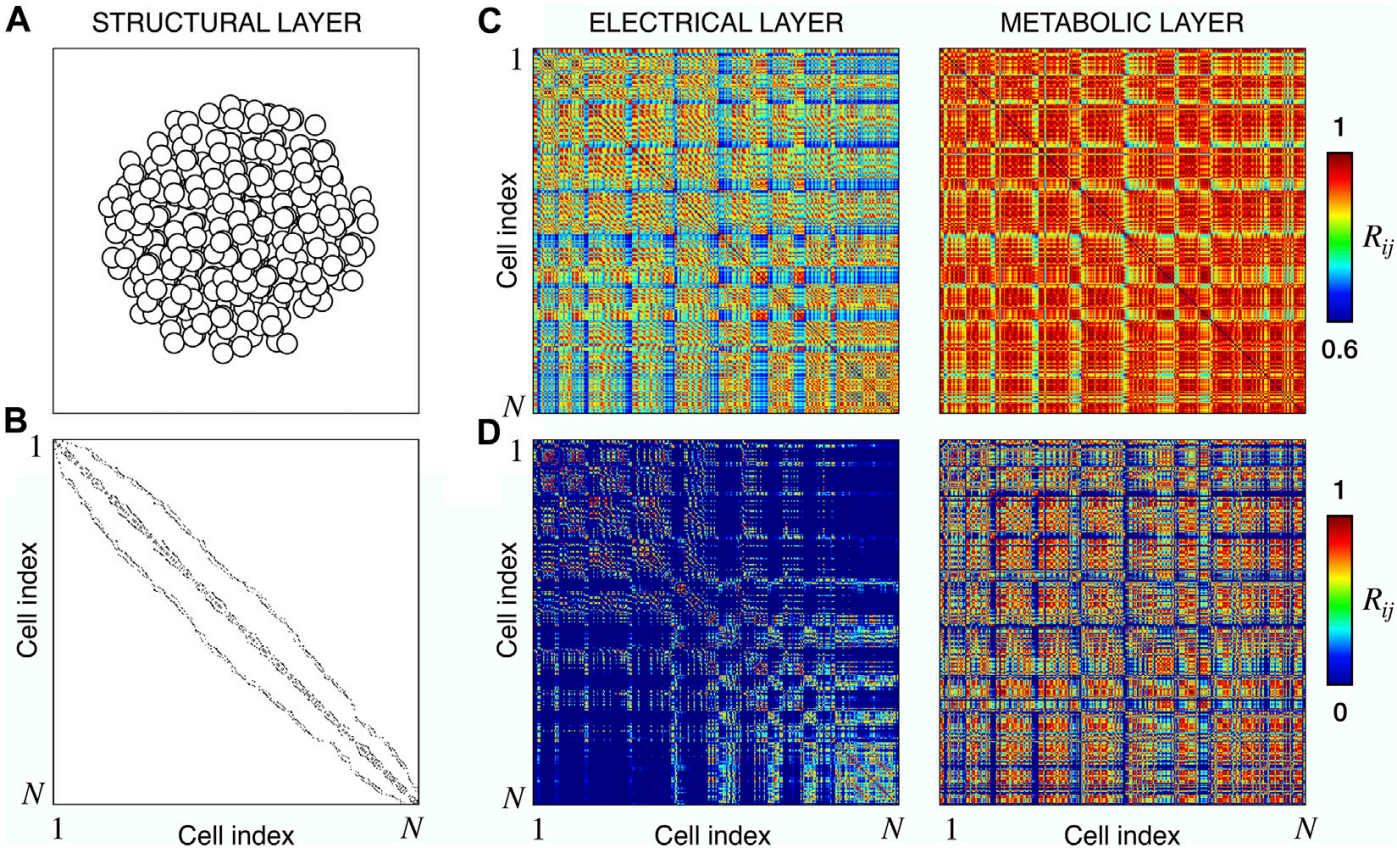
Structural connectivity and functional correlations. **(A)** Spatial arrangement of cells within the modeled islet. **(B)** Adjacency matrix of the structural *β*-cells network, obtained by connecting nearby cells as explained in Methods. **(C)** Electrical and Metabolic correlation matrices for model I1, evaluated from the membrane potential *V* and FBP binarized signals. **(D)** Same as **(C)** but for model I2. *N* denotes the total number of cells, that in our models is equal to 378.

**FIGURE 3:**
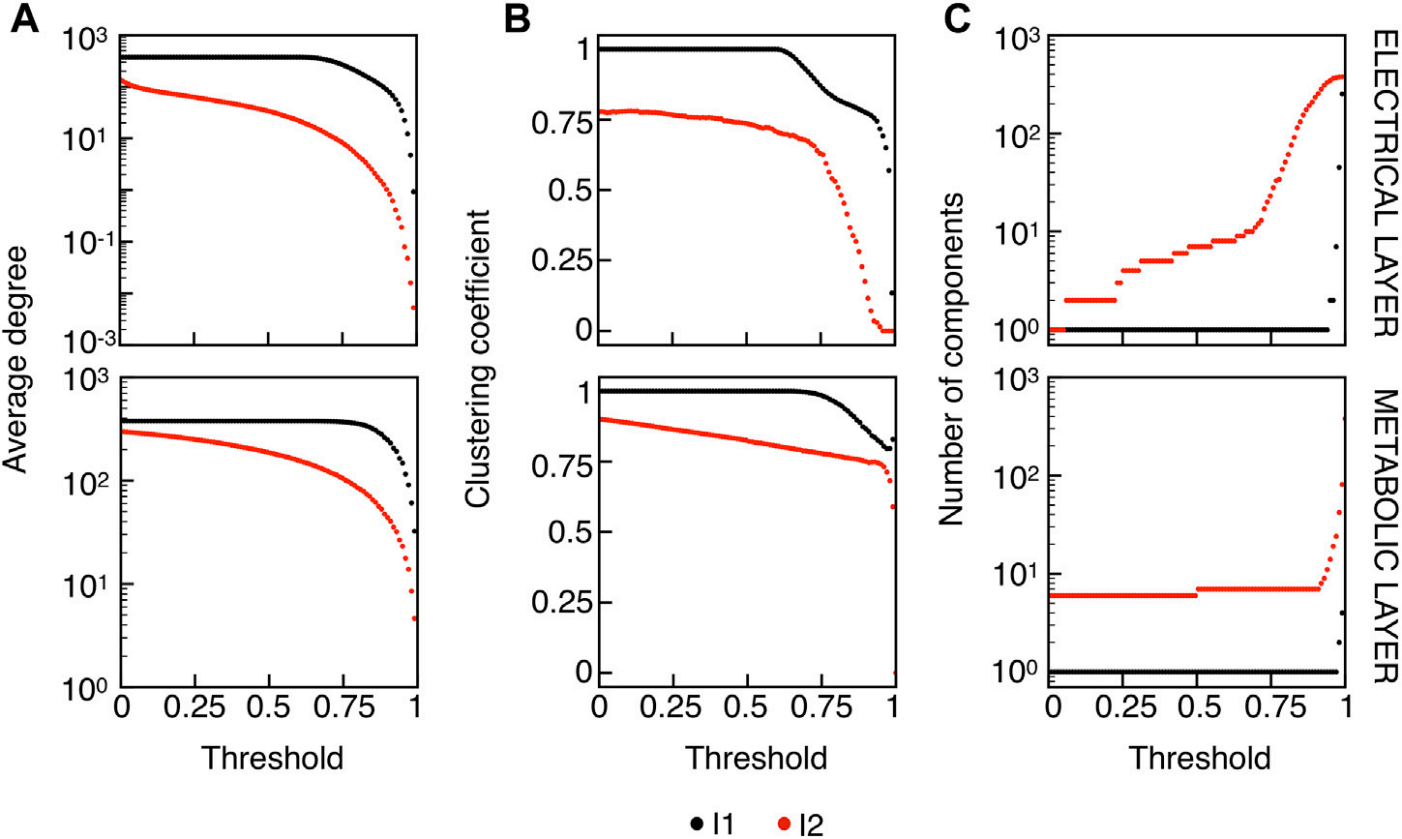
Functional networks metrics at varying thresholds. **(A)** Average node degree versus threshold. **(B)** Average local clustering coefficient versus threshold. **(C)** Number of networks connected components versus threshold. Panels on the top row show metrics for the functional electrical network, while panels on the bottom row refer to the functional metabolic network. Black dots denote values computed on model I1, and red dots on model I2. Networks efficiency (not shown), follows a similar pattern with respect to the clustering coefficient (see text).

**FIGURE 4:**
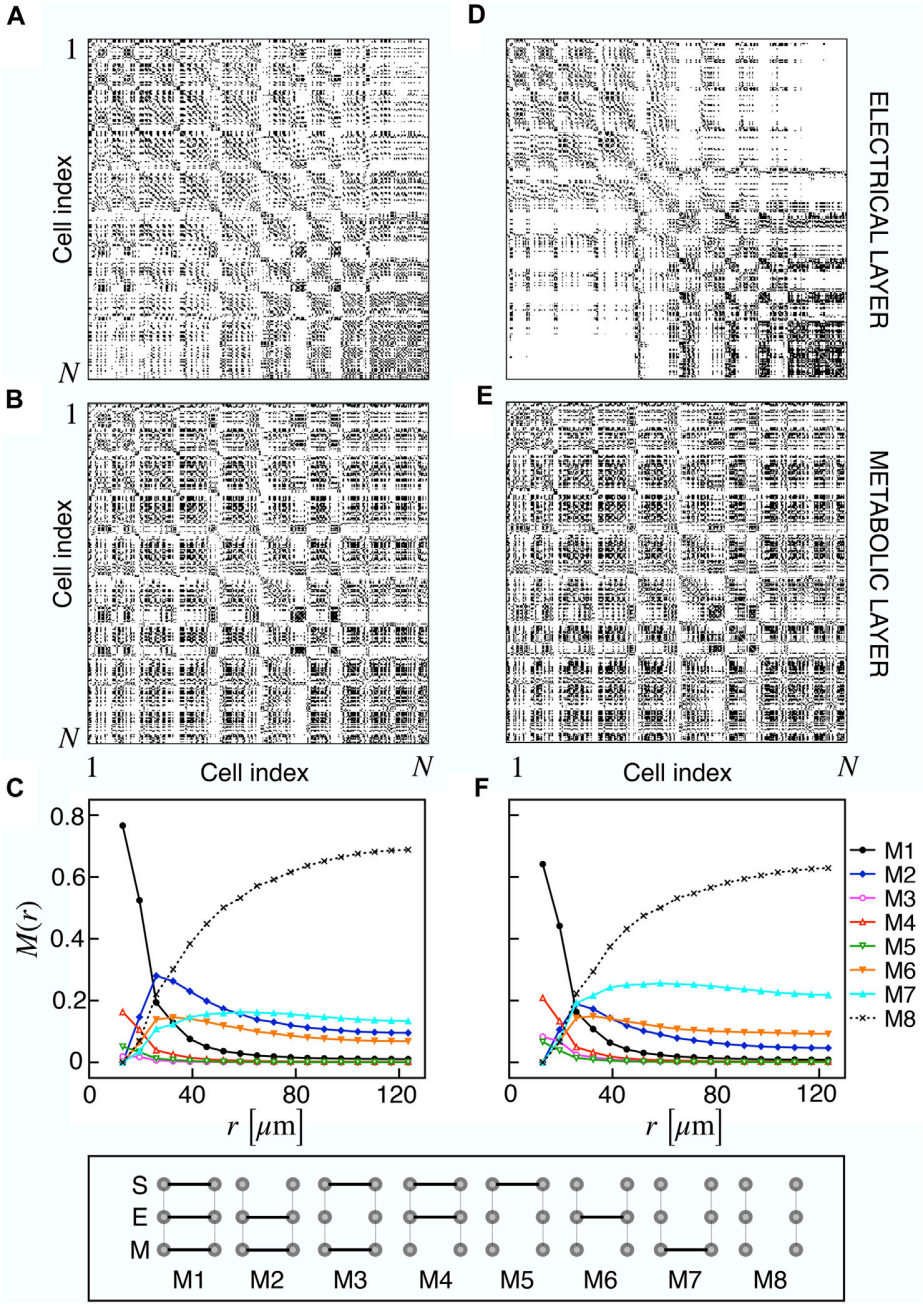
Electrical and metabolic functional connectivity matrices and multiplex motifs. **(A, B)** Electrical and metabolic functional connectivity matrices for I1, extracted with thresholds 0.9 and 0.95 for *V* and FBP, respectively. **(C)** Pairwise connection motifs frequency *M*(*r*) at increasing distances for I1. *M*(*r*) represents the relative fraction of a particular motif within the distance [0, *r*]. Considered motifs are shown in the bottom row and account for all possible connections combinations at the structural (S), electrical (E), and metabolic (M) layer: S/E/M (M1), E/M (M2), S/M (M3), S/E (M4), S (M5), E (M6), M (M7), no connections (M8). **(D, E)** Electrical and metabolic functional connectivity matrices for I2, extracted with thresholds 0.3 and 0.75 for *V* and FBP, respectively. **(F)** Pairwise connection motifs frequency *M*(*r*) at increasing distances for I2.

**FIGURE 5:**
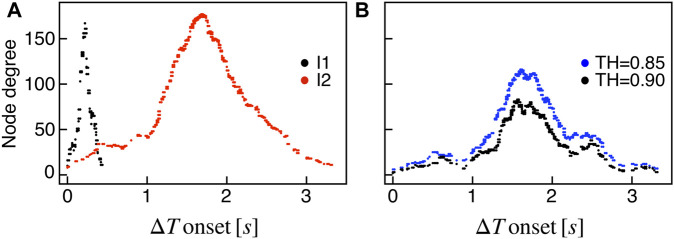
Functional nodes degree and activation wave. **(A)** Nodes degree evaluated on the metabolic functional network versus the delay of cells activation with respect to the first responding cell (Δ*T* onset, evaluated on FBP activation), for both I1 (black) and I2 (red). **(B)** Same plot as in panel **(A)** for I2, obtained by extracting the metabolic functional network at higher thresholds: R_FBP,thr_ = 0.85 (blue), R_FBP,thr_ = 0.9 (black).

We extracted functional networks from the simulated dynamics by a thresholding procedure performed on the pairwise correlation indices between binarized signals of membrane potential and FBP (see Methods). The correlation matrices evaluated from simulated signals are shown in Figure 2, in comparison to the structural adjacency matrix. In particular, the functional network is extracted by filtering only the pairwise correlations higher than a selected threshold, resulting in a binary adjacency matrix describing the functional topology (functional connectivity matrix). In order to identify the correct threshold for both the electrical layer (*V*) and the metabolic layer (FBP), we focused on the behavior of four metrics commonly used in complex networks, namely, the average degree, average local clustering, average local efficiency and number of components. Their variations versus the threshold for both I1 and I2 are shown in Figure 3. The network efficiency is not reported, having a similar variation and slightly higher values with respect to the clustering. As expected, network metrics were observed to decrease at increasing thresholds, except for the average clustering in the metabolic layer, which showed a minimal increase at thresholds close to 1. This behavior was mainly due to the fragmentation of the functional network into small connected components. When moving from 0 to 1 in terms of threshold, the functional networks range from fully connected networks (or almost fully) to fully disconnected nodes. Given its higher correlations in cells activity, the I1 model showed higher network metrics with respect to I2. To select specific thresholds for both the models and layers, we compared the metrics, minimizing the number of components, limiting the average degree to ≃ 20% of the total number of cells, and guarantying an average local clustering and local efficiency in the range 0.7–0.8 and 0.8–0.9, respectively. The aim was to avoid too sparse functional networks analyzing differences both in short- and long-range correlations between electrical and metabolic layers, therefore selecting thresholds were 0.9 and 0.95 for *V* and FBP for I1, respectively, and 0.3 (*V*) and 0.75 (FBP) for I2. Such a choice led to a relative abundance of strongly connected nodes. We also tested higher values of thresholds giving rise to more sparse networks and a reduced number of hubs, obtaining results similar to the ones here presented. The extracted functional connectivity matrices are shown in Figure 4 (panels A, B, D, and E). To analyze the link between the extracted functional networks and wave activation propagation, we checked on the relation of nodes degree with respect to Δ*T* onset for both I1 and I2 and for both FBP and *V* activations. For the membrane potential, we focused only on the first action potential wave at the active phase onset. Interestingly, for every condition, the more connected nodes were the ones activating at intermediate times (see Figure 5A). A similar bell-shaped curve was also obtained for the I2 model at higher thresholds (0.85 and 0.9 on the metabolic layer), proving that a similar relation holds in sparser functional networks (Figure 5B). In particular, activation waves in our simulations were triggered by a small peripheral population of cells with a relatively low number of functional connections, recruiting nearby connected cells, further activating inner cells, and eventually propagating the activation to the last responding cells with functional connectivity comparable to wave initiators. The occurrence of higher functional connections in intermediate activating cells can be ascribed to the fact that their activity is correlated to both first and last responders, as well as showing a high correlation among themselves. In other words, at intermediate times, a subpopulation of cells synchronously activates and delivers the activation signal from first to last responders.

Based on the extracted functional networks, the islet synchronization properties were studied by adopting a multiplex description composed of three layers: structural, electrical, and metabolic. In particular, we looked at the pairwise connection motifs in the network, analyzing their relative frequency at increasing distances from a reference cell. The analyzed motifs included all the possible connections combinations on the three layers: structural/electrical/metabolic (M1), electrical/metabolic (M2), structural/metabolic (M3), structural/electrical (M4), structural (M5), electrical (M6), metabolic (M7), disconnected (M8) (see Figure 4). Specifically, we analyzed *M*(*r*) as the relative frequency of motifs within a distance in the range [0, *r*] (Figures 4C, F). As expected, all the motifs involving a structural connection (M1, M3, M4, and M5) decreased monotonically and strongly at increasing distances for both I1 and I2. For such motifs, and consistently with the reduced value of permeability, I2 showed a reduced fraction of M1 and an increased fraction of M4, indicating a slight loss of functional metabolic coupling. Interestingly, a combined electrical and functional connection without a structural link (M2) was significantly present at distances within the range 20–50 *μ*m for both I1 and I2, indicating a significant spatial extension of combined electric and metabolic coordination. Concerning the frequency of exclusive electrical and metabolic functional connections (M6 and M7), they were found to be more abundant at *r* > 40–50 *μ*m, with long-range metabolic connections outnumbering long-range electrical ones.

### 3.3 Noise effect on synchronization motifs

**FIGURE 6:**
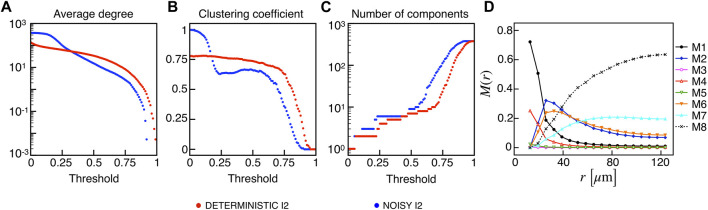
Electrical functional networks metrics and multiplex motifs in the presence of noise. **(A)** Average node degree versus threshold. **(B)** Average local clustering coefficient versus threshold. **(C)** Number of networks connected components versus threshold. The three metrics are computed on the electrical functional network for the I2 model in the presence of noise. **(D)** Pairwise connection motifs frequency *M*(*r*) at increasing distances with same thresholds used for the deterministic model I2 (Figure 4F).

Given the importance of biological noise in *β*-cells dynamics due to stochastic ion channel gating on the cell membrane, we investigated the effects of an additional stochastic term in the membrane potential dynamics on synchronization and functional multiplex motifs. In particular, we added a noise term in the membrane potential equation with shape *σW*(*t*), where *σ* represents the noise intensity, i.e., the standard deviation, and *W*(*t*) is a Gaussian white-noise process with zero mean and covariance ⟨*W*(*t*)*W*(*t*′)⟩ = *δ*(*t* − *t*′). In our simulations, we used *σ* = 0.2, and we tested the effect of such a stochastic perturbation on the I2 model, closer than I1 to the experimental observations in terms of activation waves and globally less synchronized. Interestingly, the network metrics evaluated on the binarized membrane potential revealed an increased electrical synchronization across the *β*-cells population at low thresholds (Figures 6A–C). This consideration is evident from the behavior of the average degree and number of components, indicating that the functional network exhibits a fully coupled network at very low R_V,thr_ values in these conditions. When moving to higher thresholds (≃ 0.3), the average number of connections per node is slightly higher with respect to the deterministic case (63 vs. 57), while the reduced value of the local clustering is mainly due to the appearance of locally larger coupled functional aggregates having a clustering value lower than the smaller assemblies extracted in the absence of noise. The frequency of the multiplex connection motifs (Figure 6D) nicely summarizes this increase in membrane potential synchronization, showing higher frequencies for every motif including a functional electrical connection. In this regard, combined electrical and metabolic coordination between cells pairs not structurally connected was reinforced within small groups when a moderate noise was added as input.

### 3.4 Multiple sub-populations: bimodal heterogeneity

**FIGURE 7:**
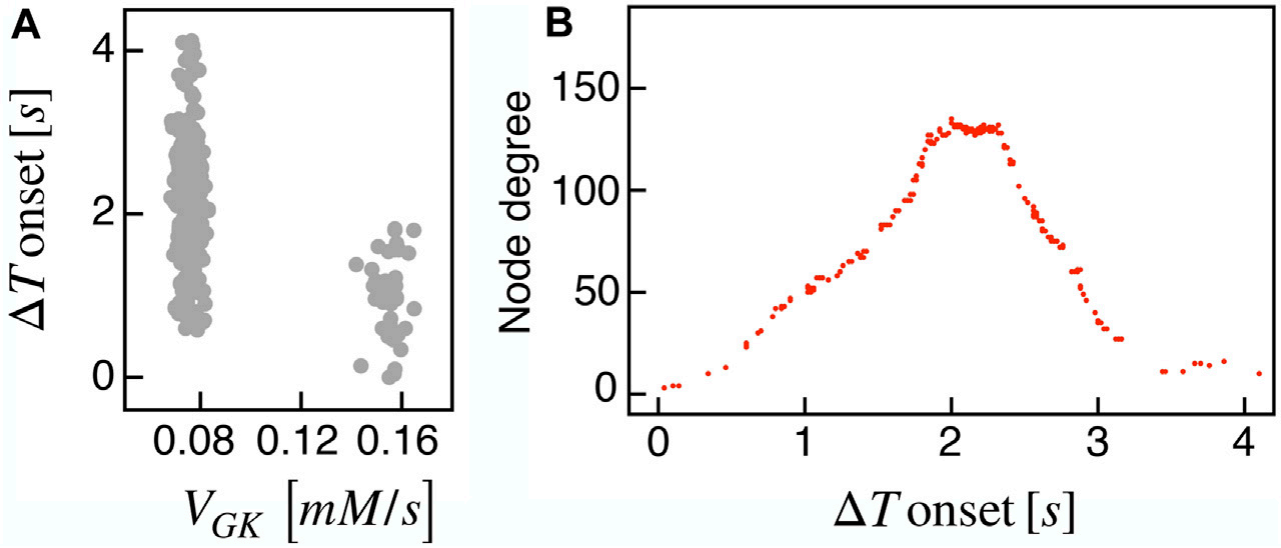
Bimodal metabolic heterogeneity and functional nodes degree in comparison to wave dynamic. **(A)** Correlation between FBP Δ*T* onset values and the glucokinase reaction rate *V*_GK_ over the *β*-cells bimodal population. **(B)** Nodes degree evaluated on the metabolic functional network versus the delay of cells activation with respect to the first responding cell (Δ*T* onset, evaluated on FBP activation). Results are computed on the I2 model.

**FIGURE 8:**
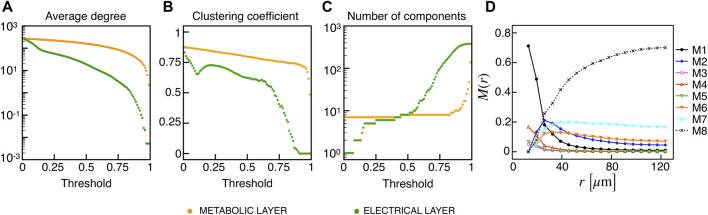
Functional networks metrics and multiplex motifs in the presence of bimodal heterogeneity. **(A)** Average node degree versus threshold. **(B)** Average local clustering coefficient versus threshold. **(C)** Number of networks connected components versus threshold. The three metrics are computed on the functional networks for the I2 model in the presence of a bimodal heterogeneity among cells, both for the electrical (green) and metabolic (orange) layers. **(D)** Pairwise connection motifs frequency *M*(*r*) at increasing distances with same thresholds used for the deterministic model I2 (Figure 4F).

Recent findings on *β*-cells network activity pointed out the existence of different sub-populations of *β*-cells. To investigate the impact of multiple sub-populations, we tested the I2 model response imposing a bimodal random sorting of the glucokinase reaction rate and defined subsets of normally responding (subset A) and highly-active cells (subset B), similarly to other modeling investigations (Dwulet et al., 2019). Specifically, we extracted the *V*_GK_ with mean and standard deviation of 0.0756 mM/s and 3% for A, and 0.1556 mM/s and 3% for B. Also, subset B included 10% of the total number of cells, in line with experimental findings on hub cells percentage in pancreatic islets, i.e., a sub-population with a characteristic fingerprint, also including increased metabolic activity. At the cell scale, slow bursting was only minimally affected, with a slight increase in bursting frequency ( ≃ 0.44 min^−1^). Also, the wave pattern activation was still present and characterized by a slight increase in time-lags between wave initiators and last-responding cells, indicating a mild decrease in the computed velocity ( ≃ 30 *μ*m/s). To address the subset of cells originating the wave, we analyzed the *V*_GK_ parameter with respect to the Δ*T* onset, as defined previously, and found that subset B was the main pacemaker of wave activity (Figure 7). No correlation between *V*_GK_ and Δ*T* onset was instead found within group A. We further analyzed functional network properties at different thresholds (Figure 8) and found no substantial differences with respect to the uni-modal case for I2 (Figure 3). At the same thresholds used for the uni-modal I2, more functionally connected cells, i.e., functional hubs, still overlapped with cells activating at intermediate time within the activation wave front (Figure 7). Further, the multiplex functional connection motifs for the bimodal case revealed correlation patterns consistent with the uni-modal model (Figure 8).

## 4 Discussion

We analyzed emergent electrical and metabolic activities in human islets by using a model with ionic currents based on biophysical measurements in human *β*-cells. The model accounted for an electric compartment fine-tuned on human electrophysiological data, a glycolytic compartment describing metabolites dynamics in *β*-cells, human-like *β*-cells networks built through hexagonal packing and ad-hoc site percolation, and nearest neighbor coupling on both membrane potential and first intermediate products of glucose metabolism (glucose 6-phosphate and fructose 6-phosphate). We tested the model in a slow metabolically-driven bursting mode with a fixed electrical coupling conductance of 0.01 nS/pF, a value in line with previous estimates on nearby human *β*-cells, and sufficiently high to guarantee electrical synchronization in small compact cells’ aggregates (Loppini et al., 2015; Loppini et al., 2017). As for the metabolites’ permeability, we restricted our simulations to two coupling values, i.e., *P*_G6PF6P_ = 0.001, 0.01 ms^−1^, with the higher value consistent with a previous estimation on coupled mouse *β*-cells (Kohen et al., 1979; Loppini et al., 2015). We found that both settings resulted in similar features of spacetime dynamics, with the same slow bursting time scale at the single-cell level and wave propagation phenomena at bursting onset, present on membrane potential, intracellular calcium, and fructose 1,6-bisphosphate FBP. Wave velocity varied significantly with metabolites’ permeability, and we were able to match experimental observations (Gosak et al., 2022), reporting velocities of ≃ 10 *μ*m/s, only at *P*_G6PF6P_ = 0.001 ms^−1^. These findings suggest that gap-junction permeability to metabolites can support slow wave propagation in human islets, and its value could be significantly lower than the value estimated for the mouse. Previous studies focusing on waves propagation in mouse islets and their modeling with pure electrical coupling pointed out higher velocities in the mouse, ranging from 50 to 100 *μ*m/s, that could be numerically reproduced with heterogeneous distributions of single-cell parameters and cells coupling strengths, or by enhancing KATP channels conductance (Aslanidi et al., 2001; Benninger et al., 2008; Stožer et al., 2013a; Šterk et al., 2021). In these regards, metabolic coupling can serve as an alternative or additional mechanism through which slow activation waves can propagate across pancreatic islets. It is worth noting that also in human islets connexin 36 expression variability and corresponding heterogeneities in coupling strengths can impact cell synchronization and potentially shape activation wave velocity. In this regard, future studies should explore this aspect, addressing its role in the collective dynamics of human *β*-cell networks.

In our simulations, first wave-initiators were also the cells with higher glucokinase activity, while in multi-species experimental observations combining imaging and transcriptomics (Johnston et al., 2016; Salem et al., 2019) increased metabolic activity was only proven in hub cells. In this regard, our results can be viewed as a model prediction of pacemakers metabolic fingerprint in human islets showing metabolically-driven slow bursting oscillations. Consistently with other experimental analyses on mouse islets (Šterk et al., 2023b), the pacemakers identified in our analysis were mostly located in the islet periphery and characterized by a reduced number of structural and functional connections. In line with Šterk et al. (2023b), our functional networks showed a reduced number of links for wave initiators and terminators (last responding cells) and an increased number of connections for intermediate responding cells (Figures 5, 7). Indeed, our results confirm that functional hubs are cells spreading activity from the wave-initiators to the rest of the *β*-cells population and can naturally arise because of a spacetime activation supported by nearest-neighbor structural couplings (Cappon and Pedersen, 2016; Šterk et al., 2023b).

In the domain of functional networks, our simulations show interesting features of multi-level synchronization in *β*-cells aggregates. Our results reveal that at *P*_G6PF6P_ = 0.01 ms^−1^ the extracted functional networks are characterized by too dense connectivities in comparison to experimental observations (Gosak et al., 2022), further corroborating that low metabolic coupling strengths can be implied in the regulation of human *β*-cell networks, in line to what was pointed out by the wave velocity analysis. Given the frequency of the functional motifs versus distance, human *β*-cells networks are able to support combined electrical and metabolic coordination that extend at longer ranges with respect to the underlying structural connectivity. Indeed, both electrical and metabolic functional links were found within distances from a reference cell of about 20–50 *μ*m, supporting the idea of significant coordination of cells activity in small groups (Quesada et al., 2006; Gosak et al., 2022). At low metabolites permeabilities, such coordination is slightly less strong but still significant. At longer distances, metabolic coordination overcomes electrical one, suggesting that metabolic dynamics could be less sensitive to cells spatial aggregation in small groups within human islets. A possible explanation could be that the slow glycolytic oscillators are more prone to synchronization than the fast electrical ones or that heterogeneity in glycolytic parameters is as such to ensure longer range coordination. Our results are in agreement with a recent study on combined electrical and metabolic coupling in *β*-cells networks based on a phenomenological model reproducing fast and slow oscillations (Šterk et al., 2023a). Specifically, Šterk et al. (2023a) showed that functional networks derived from the slow metabolic activity present more long-range connections than the ones extracted from the fast electrical activity and that metabolic coupling can boost synchronization of the fast electrical oscillations. Consistently, the motifs analysis here presented showed an increased fraction of functional connections on the electrical layer at high metabolic coupling strengths and, in general, metabolic synchronization spanning longer distances than the electrical one. It is worth mentioning that we also tested other techniques to extract functional networks based on the original simulated signals or on their derivatives, and we obtained results in line with the one presented here obtained with binarized time series describing cells activation (see Methods). The only parameter that changes in these different approaches is the threshold used to extract significant correlations (Gosak et al., 2018). An intriguing further result is that electrical synchronization appeared to be enhanced when randomness is included in the model in the form of white noise within the membrane potential dynamics. It is well known that noise deriving from stochastic ion channel gating is particularly significant in *β*-cells, and when included in coupled *β*-cell populations, it can potentiate bursting oscillation and induce stochastic resonance phenomena (Sherman and Rinzel, 1991; De Vries and Sherman, 2000). Our results show that similar stochastic resonance phenomena, in relation to action potentials synchronization, can potentially enhance electrical coordination in human *β*-cells aggregates.

Concerning the impact of different cell sub-populations, we verified that a bimodal distribution of *V*_GK_ resulted in non significant variations of wave-pattern activation and synchronization patterns, further confirming the role of cells with increased *V*_GK_ as wave initiators. These results are in apparent contradiction with experiments and simulations made in other studies on mouse *β*-cell aggregates (Westacott et al., 2017), which showed that wave initiators had altered excitability, as stated also in Benninger et al. (2008), Benninger et al. (2014), and were less metabolically active. Concerning simulations, this contradiction can be partially mitigated if it is analyzed the effect of lowering and increasing *V*_GK_ in our model with respect to the one reported by Westacott et al. (2017). Indeed, increasing *V*_GK_ in our model results in an increasing metabolic oscillations frequency, which is the opposite effect compared to the one observed in ref. (Westacott et al., 2017), where it is obtained an increasing frequency at lower *V*_GK_ values. Therefore, wave initiators in the two models consistently show higher intrinsic frequencies, in line with what could be expected in diffusively coupled networks of nonlinear oscillators. It is worth noting that the *β*-cell glycolytic oscillator on which we based our network model (Westermark and Lansner, 2003) presents a U-shaped behavior in terms of oscillation period versus *V*_GK_, and moving the extraction of such a parameter to higher values could reproduce frequency increasing by lowering the glucokinase reaction rate. Other than that, other major factors can explain the observed differences. Indeed, we here study slow metabolically-driven bursting, while Westacott et al. (2017) investigated a fast electrically-driven bursting. In this regard, our model discards metabolic oscillations driven by the electrical compartment, which, in some conditions, have been shown to be dominant oscillation modes in a single-cell model fine-tuned on mouse data (Marinelli et al., 2021). Further refinements of the electro-metabolic feedback to the human case should be needed to test such oscillations and their synchronization properties in human islets. Also, our study focuses on human *β*-cells networks, and their collective response can differ from the one observed in the mouse. Additional experiments based on a more precise quantification of metabolic activity could help in dissecting both differences and similarities between the mouse and the human. Still, in our bimodal model, functional hubs did not overlap with wave initiators and cells presenting altered *V*_GK_ values. To summarize, our results can be interpreted as a prediction of increased metabolic activity for wave initiators in human *β*-cell aggregates regulated in a slow metabolic bursting regime. Further studies should be devoted in this context to verify this result by extracting comprehensive fingerprints of initiators and hubs, also involving detailed electrophysiological characterizations.

In conclusion, this study revealed interesting properties of human-like *β*-cells networks and pointed out that metabolic coupling can be an important mechanism regulating whole-islet activity in humans and, potentially, also in the mouse and in other species. The multi-level analysis of cells activity correlation also showed that functional architecture reveals more complexity than what is merely seen when looking at the sole electrical/calcium dynamics. Furthermore, functional metabolic networks can give additional valuable insights on the coordination properties of *β*-cells networks and their possible impairment in pathology. Future studies should be devoted to addressing some limitations of the approach presented here, among which heterogeneity in electrical gap-junction conductance and permeability, electro-metabolic coupling, large islet architectures, and the inclusion of *α*- and *δ*-cells together with heterotypic cells interaction, as previously performed by Briant et al. (2018), pointing toward the modeling of realistic whole-islet activity.

## Data Availability

The raw data supporting the conclusion of this article will be made available by the authors, without undue reservation.
